# Pregnancies in Women With Kidney Failure on Home Dialysis in the United States

**DOI:** 10.1016/j.ekir.2024.01.045

**Published:** 2024-02-01

**Authors:** Silvi Shah, Eric Weinhandl, Anthony C. Leonard, Brenna Rachwal, Prasoon Verma, Jeffrey Perl, Annette L. Christianson

**Affiliations:** 1 Division of Nephrology Kidney C.A.R.E. Program, University of Cincinnati, Cincinnati, Ohio, USA; 2 Satellite Healthcare, San Jose, California, USA; 3 Department of Pharmaceutical Care and Health Systems, College of Pharmacy, University of Minnesota, Minneapolis, Minnesota, USA; 4 Department of Environmental Health, University of Cincinnati, Cincinnati, Ohio, USA; 5 Division of Neonatology, Cincinnati Children’s Hospital Medical Center, Cincinnati, Ohio, USA; 6 St. Michael's Hospital, University of Toronto, Ontario, Canada

**Keywords:** home hemodialysis, kidney failure, peritoneal dialysis, pregnancy, rates

## Abstract

**Introduction:**

Women with kidney failure have impaired fertility and are at a higher risk of maternal and fetal morbidity and mortality. Little is known about pregnancies in women receiving maintenance home dialysis in the United States.

**Methods:**

Using data from the United States Renal Data System (USRDS), a cohort of 26,387 women aged 15 to 49 years with kidney failure receiving maintenance home dialysis from 2005 to 2018 was examined. We calculated pregnancy rates and identified factors, including the modality associated with pregnancy receiving home dialysis.

**Results:**

Overall, 437 pregnancies were identified in 26,837 women on home dialysis. The unadjusted pregnancy rate was 8.6 per 1000 person-years (PTPY). The unadjusted pregnancy rate was higher on home hemodialysis (16.0 vs. 7.5 PTPY) than on peritoneal dialysis. Women receiving home hemodialysis had a higher adjusted likelihood of pregnancy than women receiving peritoneal dialysis (hazard ratio [HR], 2.34; 95% confidence interval [CI], 1.79–3.05). Compared with women aged 20 to 24 years, the likelihood of pregnancy was lower in women aged 30 to 34 years (HR, 0.64; 95% CI, 0.43–0.96), 35 to 39 years (HR, 0.53; 95% CI, 0.35–0.79), 40 to 44 years (HR, 0.32; 95% CI, 0.21–0.49), and 45 to 49 years (HR, 0.21; 95% CI, 0.13–0.33). Whereas Black women had a higher likelihood of pregnancy (HR, 1.40; 95% CI, 1.07–1.83), there was no difference in likelihood of pregnancy in Asian, Hispanic, and Native Americans as compared to Whites. Body mass index, cause of kidney failure, socioeconomic status, rurality, predialysis nephrology care, or dialysis vintage were not significantly associated with pregnancy on home dialysis.

**Conclusion:**

The pregnancy rate in women with kidney failure undergoing home dialysis is higher with home hemodialysis than with peritoneal dialysis. Younger age and Black race or ethnicity are associated with a higher likelihood of pregnancy among women receiving home dialysis. This information can guide clinicians in preconception counselling and making informed treatment decisions for pregnant women on home dialysis.

## Graphical abstract

**Figure:**
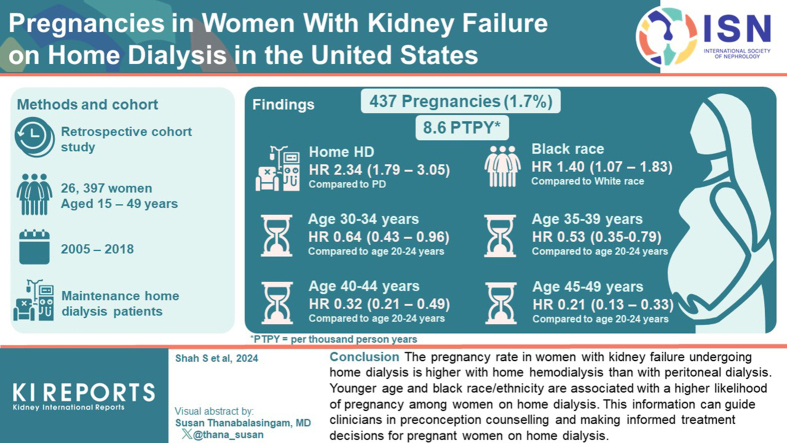

See Commentary on Page 746

Pregnancy in women with kidney failure is not uncommon^1^, ^2^, ^3^ Women with kidney failure have reduced fertility due to disruption of the hypothalamic gonadal axis, anovulation, and hyperprolactinemia.^4^ Pregnancy is further challenging in women with kidney disease due to a higher risk of maternal and fetal morbidity and mortality, and drugs such as mycophenolate used to treat kidney disease such as lupus are contraindicated due to their teratogenicity risk.^5^^,^^6^

Currently, in the United States, the prevalence of home dialysis use is 13.1%, with 1.9% performing home hemodialysis and 11.2% performing peritoneal dialysis.^7^ The Executive Order on Advancing American Kidney Health emphasizes the use of home dialysis therapies, specifically home hemodialysis and peritoneal dialysis, due to the convergence of clinical evidence that supports home-based therapies.^8^ The utilization of home dialysis is even more critical for women of childbearing age due to the importance of mother-child bonding during and after pregnancy.^9^

Although several studies have reported pregnancy rates and outcomes in women undergoing in-center hemodialysis, there is little information on a global level about pregnancy and childbirth in women with kidney failure treated with home dialysis.^2^^,^^3^^,^^10^ Existing estimates for home dialysis pregnancies are likely biased from experienced centers and mostly rely on case reports, case series, voluntary surveys, or single-center studies.^11^, ^12^, ^13^, ^14^ In addition, the small number of cases precludes our understanding of the factors associated with the likelihood of pregnancy on home dialysis. With the increase in the use of home-based dialysis therapies in the United States and an increasing focus on women’s health, closing this critical knowledge gap becomes particularly important for clinicians and policymakers to help with preconception counseling, shared decision-making, and inform treatment decisions during pregnancy.

The objectives of this study were to determine pregnancy rates on home dialysis and investigate the association of treatment modality (home hemodialysis and peritoneal dialysis) and other demographic and clinical characteristics with the occurrence of pregnancy among women undergoing home dialysis in the United States.

## Methods

### Data Sources and Study Population

**Figure 1:**
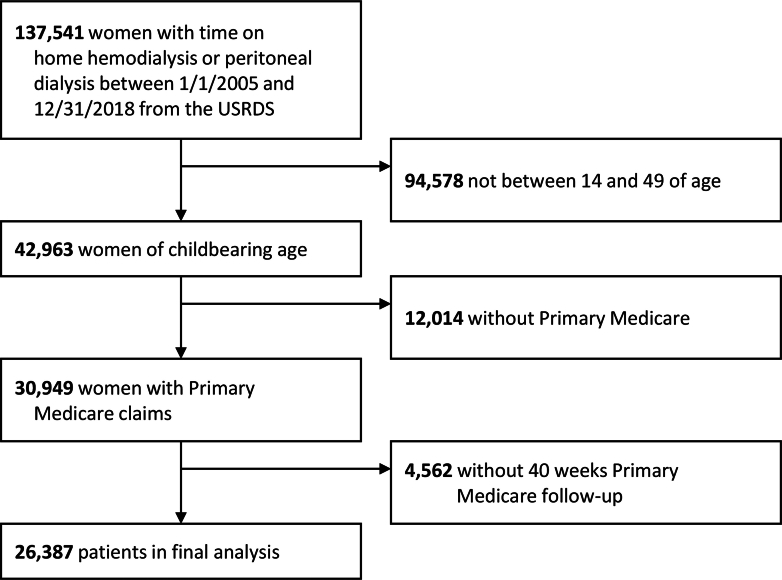
Cohort selection flow diagram. USRDS, United States Renal Data System.

The data came from the USRDS, which is the national registry of patients undergoing maintenance dialysis or having received a kidney transplant.^7^ We performed a retrospective cohort study of 26,387 female patients with kidney failure who had any days with the following criteria simultaneously: on home dialysis, aged 15 to 49 years, with Medicare as their primary payer and 40 weeks of additional uninterrupted follow-up, and between January 1, 2005, and December 31, 2018. Person-time at risk for the outcome of pregnancy included all days which satisfied all criteria; it was possible for a female to have multiple such periods, in which case all periods were included in time at risk. Patient time-at-risk ended when a woman turned 50 years old, died, received a kidney transplant, stopped home dialysis, or 40 weeks before the end of her primary Medicare coverage, or on December 31, 2018. The 40-week follow-up time period was used only to identify pregnancy-related codes; it required survival but could continue after transplant, 50th birthday, or discontinuation of home dialysis. In Figure 1, we illustrate the study cohort derivation. The study was deemed exempt by the University of Cincinnati Institutional Review Board Committee due to the use of deidentified data.

### Outcome Ascertainment

Pregnancy in women undergoing home dialysis was the primary outcome of interest. Pregnancy was identified by the International Classification of Diseases, Ninth Revision, Clinical Modification (ICD-9-CM*)* codes; International Classification of Diseases, 10th Revision, Clinical Modification (ICD-10-CM*)* codes; Current Procedural Terminology, Fourth revision codes and diagnostic related group codes. The method was adapted from a validated method^10^ and used in previous reports.^3^^,^^15^ For this study, the only changes were to add ICD-10-CM codes using general equivalence mappings and add updated versions of the diagnostic related group codes.

All codes were used to construct pregnancy episodes, in order to identify separate pregnancies within the study period without overcounting the pregnancy episodes. Codes that marked the end of pregnancy were considered to be most accurate with regard to timing and fetal outcome and were used in the following order: ICD-9/ICD-10 diagnosis codes denoting live births; ICD-9/ICD-10 procedure codes indicating end of pregnancy; Current Procedural Terminology, Fourth Revision procedure codes indicating end of pregnancy; diagnostic related group codes which showed reasons for hospitalization related to end of pregnancy; other ICD-9/ICD-10 diagnosis codes denoting end of pregnancy; and other ICD-9/ICD-10 codes denoting current pregnancy. Additional codes during the identified pregnancies or within 8 weeks were used only to update information about fetal outcomes when possible. The fetal outcome was identified by codes within the gestation period, with the following hierarchy: live birth and stillbirth (in twin pregnancies), live birth, ectopic or trophoblastic pregnancy, stillbirth, spontaneous abortion, and therapeutic abortion (discharge diagnoses and medical procedures indicative of pregnancy are in the Supplementary Material). Outcome-specific estimates of gestational age were used to estimate conception dates as follows: deliveries not identified as stillbirths or multiple, 40 weeks; twins, 36 weeks; triplets, 33 weeks; quadruplets, 31 weeks; stillbirths, 28 weeks; all abortions, 10 weeks; and ectopic or trophoblastic pregnancies, 8 weeks. The conception date for twin pregnancies resulting in both live birth and stillbirth was estimated at 40 weeks. Further codes that demonstrated the presence of pregnancy but not the timing or outcome were then grouped together to identify pregnancies with unknown outcomes. Unidentified early losses that occurred within 6 months of the end of a previous pregnancy other than a live birth were deleted. Finally, the remaining pregnancies were examined for consistency of dates. When overlapping pregnancy episodes were identified, a review was conducted to determine whether to delete one or adjust the date of the second pregnancy episode.

An interval of at least 24 weeks was required between the ends of 2 pregnancies resulting in deliveries, 20 weeks between an early loss and a subsequent delivery, 10 weeks between a delivery and a subsequent early loss, and 6 weeks between 2 early losses. Start dates for pregnancies that resulted in delivery were adjusted by a maximum of 8 weeks in case of overlap. Start dates for pregnancies that resulted in early loss were adjusted by a maximum of 2 weeks.^15^, ^16^, ^17^, ^18^ As the interest was in pregnancies initiated while on home dialysis, the imputed conception date was used as the event date for all survival analyses and rates. In Supplementary Figure S1, we show the stepwise approach of the methodology to identify pregnancies.

### Exposures of Interest and Covariates

Home dialysis modality (home hemodialysis or peritoneal dialysis) at the time of each conception was determined from the USRDS treatment history files. Changes in modality according to the file led to either updated modality or end of person-time at risk. Gaps of fewer than 30 days between intervals of home hemodialysis were ignored; thus, that time was attributed to home hemodialysis.

Patient-level information, including birth date, race or ethnicity, cause of end-stage kidney disease, date of onset of end-stage kidney disease, body mass index, and previous nephrology care, were obtained from the Centers for Medicare and Medicaid Services End-Stage Renal Disease Medical Evidence Report (Form CMS-2728) recorded at the time of end-stage kidney disease registration.^19^ Information on dialysis dates was obtained from the USRDS treatment history files. The USRDS residence file provided zip codes of patient's residence at study entry.

Race or ethnicity was categorized as non-Hispanic Asian, non-Hispanic Black, Hispanic, non-Hispanic Native American, non-Hispanic White, and others/unknown. The history of previous nephrology care was grouped into none, ≤12 months, >12 months, and unknown. Age was updated based on birthdays; dialysis vintage (years spent on dialysis; <1 year, 1–3 years, and >3 years) was updated based on dialysis treatment records. Cause of end-stage kidney disease was categorized as cystic/hereditary/congenital, diabetes mellitus, glomerulonephritis, hypertension/large vessel disease, interstitial nephritis/pyelonephritis, malignancy, secondary glomerulonephritis/vasculitis, and others. Zip codes were combined with zip code level data from the United States Census Bureau American Community Survey 5-year estimates from 2007 to 2011 to determine neighborhood socioeconomic status, which was defined as the percent of zip code residents living below the federal poverty level and grouped similarly to the United States Census Bureau literature into 5 categories as follows: I (<13.8%), II (13.8%–19.9%), III (20.0%–39.9%), IV (40% or more), and unknown. The rurality of the neighborhood was determined using the rural-urban commuting area code version 2.0 and grouped into 4 categories as follows: metropolitan (rural-urban commuting area 1.0–3.9), micropolitan (rural-urban commuting area 4.0–6.0), rural (rural-urban commuting area 7.0–10.6), and unknown.^20^^,^^21^ Groups for body mass index were created based on clinical relevance with patients with unavailable information on covariates categorized into a “missing” group, as shown in Table 1.

**Table 1:** Characteristics of women with kidney failure on home dialysis separated by pregnancy status

Characteristics	Overall (*N* = 26,387)	Never pregnant (*N* = 25,976)	Ever pregnant (*N* = 411)	*P* value
Type of dialysis modality				<0 .0001
Home hemodialysis	11.6	11.5	19.7	
Peritoneal dialysis	88.4	88.5	80.3	
Race/ethnicity				0.0004
Asian	4.0	4.0	2.9	
Black	38.9	38.7	47.9	
Hispanic	14.8	14.8	17.3	
Native American	1.3	1.3	∗	
White	39.7	39.8	29.9	
Unknown/others	1.3	1.3	∗	
Age (yr)^a^	37 (9)	38 (9)	33 (8)	<0 .0001
				<0 .0001
15–19	3.8	3.8	5.4	
20–24	5.7	5.6	12.2	
25–29	10.2	10.1	17.3	
30–34	14.0	13.8	24.6	
35–39	17.7	17.7	19.2	
40–44	22.2	22.3	14.6	
45–49	26.3	26.7	6.8	
Cause of kidney failure				<0 .0001
Diabetes mellitus	27.3	27.5	18.0	
Glomerulonephritis	22.2	22.1	28.5	
Secondary glomerulonephritis/vasculitis	12.7	12.6	16.1	
Interstitial nephritis/pyelonephritis	3.7	3.8	∗	
Hypertension/large vessel disease	18.4	18.4	17.3	
Cystic/hereditary/congenital	7.2	7.2	6.1	
Malignancy	0.7	0.7	∗	
Other	7.7	7.7	10.5	
Prior nephrology care				0.6800
None	15.2	15.3	14.6	
≤ 12 months	25.3	25.3	23.8	
> 12 months	20.8	20.8	20.0	
Unknown	38.7	38.6	41.6	
Body mass index (kg/m^2^)	29.6 (8.9)	29.6 (8.9)	29.3 (8.9)	0.4274
				0.8360
< 18.50	5.2	5.2	5.6	
18.50–24.99	27.3	27.3	27.3	
25.00–29.99	20.5	20.5	21.9	
≥ 30.00	38.3	38.3	35.8	
Missing	8.7	8.7	9.5	
Dialysis vintage (years)	2.2 (3.0)	2.2 (3.0)	2.3 (3.2)	0.3901
				0.5355
< 1 year	46.4	46.4	44.8	
1–3 years	31.3	31.3	30.7	
> 3 years	22.3	22.3	24.6	
Neighborhood poverty level				0.7236
< 12.8%	55.5	55.5	52.3	
12.8%–19.9%	20.2	20.1	22.1	
20%–39.9%	21.2	21.2	22.6	
≥ 40%	1.8	1.8	∗	
Unknown	1.4	1.4	∗	
Neighborhood rurality				0.1074
Metropolitan	76.5	76.4	81.3	
Micropolitan	10.7	10.8	9.5	
Rural	10.8	10.8	∗	
Unknown	2.0	2.0	∗	

Age, dialysis modality, dialysis vintage, poverty, and rurality were determined at study entry; other covariates were determined at end-stage kidney disease initiation.

a Reported in mean (standard deviation), all others reported in percentages.

∗ Per Centers for Medicare and Medicaid cell suppression policy, cells that display individuals (n) from 1 to 10 or cells that allow a value of 1 to 10 derived from other reported cells are suppressed.

### Statistical Analysis

Patient characteristics were described using mean and SD for continuous variables and count and percent for categorical variables. Differences between groups were studied using chi-square tests for categorical variables and t-tests or 1-way analysis of variance for continuous variables. These comparisons did not account for person-time at risk or multiple pregnancies and used characteristics at study entry for the time-dependent covariates of age, dialysis modality, and dialysis vintage. Statistical significance was set at a 2-tailed *P*-value of 0.05, unadjusted for multiple tests. Unadjusted pregnancy rates were expressed as number of pregnancies PTPY, with CIs calculated using the Poisson distribution.

Due to the possibility of multiple pregnancies per woman, we used the Prentice, Williams, and Peterson total time recurrent event time-to-event analysis, with sandwich variance estimators, to evaluate associations of home dialysis modality and other patient characteristics with pregnancy.^22^ The Prentice, Williams, and Peterson model stratifies for repeat pregnancies to allow hazards to differ for subsequent events. Due to the small number of repeat pregnancies, we used only 2 strata, for initial and repeat pregnancies. Multivariable time-to-event models were nonparsimonious, and time under observation was censored at the end of the person’s time at risk. The final time-to-event models included baseline covariates of race/ethnicity; cause of end-stage kidney disease; previous nephrology care; body mass index; poverty; rurality; and the time-dependent covariates of age, dialysis modality, and dialysis vintage. The covariates for adjusted analyses were chosen based on their known clinical relevance. The risk estimates were expressed as HRs and their 95% CIs. All data were analyzed using SAS 9.4 (SAS Institute, Cary, NC).

## Results

### Baseline Demographics and Clinical Characteristics

Overall, 437 pregnancies were identified in 26,387 women on home dialysis. In Table 1, we show the characteristics of women receiving home dialysis with pregnancies and those who did not become pregnant. The mean age was 37 ± 9 years, and women who conceived were younger as compared to women who did not conceive (33 ± 8 years vs. 38 ± 9 years). 19.7% of the women who conceived received home hemodialysis, as compared to 11.5% of women who did not conceive. Black race or ethnicity was more likely among those who conceived on home dialysis (47.9% vs. 38.7%). Glomerulonephritis was the most common cause of kidney failure (28.5%) among women who conceived, whereas diabetes was the most common cause of kidney failure among women who did not conceive (27.5%). There were no significant differences in body mass index, previous nephrology care, dialysis vintage, neighborhood poverty, or neighborhood rurality between women who conceived and those who did not conceive.

In Table 2, we show the characteristics of pregnancies on home dialysis separated by modality at conception. The mean age of women who conceived on home dialysis was 34 ± 8 years. Highest proportion of pregnancies on home hemodialysis and peritoneal dialysis were in the women aged 30 to 34 years, and 35 to 39 years, respectively. Mean body mass index was higher for women with pregnancies on home hemodialysis (30.9 ± 9.9 kg/m^2^ vs. 28.9 ± 8.8 kg/m^2^). Mean dialysis vintage was 4.3 ± 3.8 years, with pregnancies on home hemodialysis having a longer time on dialysis as compared to pregnancies on peritoneal dialysis (6.8 ± 4.8 years vs. 3.5 ± 3.0 years).

**Table 2:** Characteristics of pregnancies in women with kidney failure receiving home dialysis separated by modality at conception

Characteristics	Overall (*N* = 437)	Home hemodialysis (*N* = 109; 24.9%)	Peritoneal dialysis (*N* = 328; 75.1%)	*P* value
Race/ethnicity				0.0429
Asian	2.7	∗	∗	
Black	48.5	53.2	47.9	
Hispanic	16.7	∗	19.5	
Native American	∗	∗	∗	
White	30.0	34.9	28.4	
Unknown/others	∗	0.0	∗	
Age (years)^a^	34 (8)	35 (7)	34 (8)	0.8069
				0.0484
15–19	2.5	∗	∗	
20–24	9.4	∗	∗	
25–29	15.8	∗	18.0	
30–34	21.3	30.3	18.3	
35–39	22.9	26.6	21.6	
40–44	15.6	15.6	15.5	
45–49	12.6	∗	13.7	
Cause of kidney failure				0.0187
Diabetes mellitus	17.2	∗	20.1	
Glomerulonephritis	28.6	34.9	26.5	
Secondary glomerulonephritis /vasculitis	15.8	11.9	17.1	
Interstitial nephritis/pyelonephritis	3.2	∗	∗	
Hypertension/large vessel disease	18.3	25.7	15.9	
Cystic/hereditary/congenital	5.7	∗	6.4	
Malignancy	∗	0.0	∗	
Other	∗	12.8	10.1	
Prior nephrology care				<0.0001
None	14.0	10.1	15.2	
≤ 12 months	23.3	17.4	25.3	
> 12 months	19.2	10.1	22.3	
Unknown	43.5	62.4	37.2	
Body mass index (kg/m^2^)^a^	29.4 (9.1)	30.9 (9.9)	28.9 (8.8)	0.0680
				0.0005
< 18.50	5.7	∗	7.0	
18.50–24.99	26.5	25.7	26.8	
25.00–29.99	21.3	13.8	23.8	
≥ 30	35.7	38.5	34.8	
Missing	10.8	∗	7.6	
Dialysis vintage (yr)^a^	4.3 (3.8)	6.8 (4.8)	3.5 (3.0)	<0.0001
				<0.0001
< 1 year	29.5	∗	33.2	
1–3 years	16.5	∗	19.8	
> 3 years	54.0	75.2	47.0	
Neighborhood poverty level				0.4201
< 12.8%	53.3	57.8	51.8	
12.8%–19.9%	20.1	14.7	22.0	
20%–39.9%	23.1	22.9	23.2	
≥ 40%	∗	∗	∗	
Unknown	∗	∗	∗	
Neighborhood rurality				0.7566
Metropolitan	81.2	84.4	80.2	
Micropolitan	8.7	∗	9.5	
Rural	8.2	∗	∗	
Unknown	∗	∗	∗	

Age, dialysis modality, and dialysis vintage were determined at conception; poverty and rurality were determined at study entry; other covariates were determined at end-stage kidney disease initiation.

a Reported in mean (standard deviation), all others reported in percentages.

∗ Per Centers for Medicare and Medicaid cell suppression policy, cells that display individuals (n) from 1 to 10 or cells that allow a value of 1 to 10 derived from other reported cells are suppressed.

### Unadjusted Rates of Pregnancies on Home Dialysis

**Figure 2:**
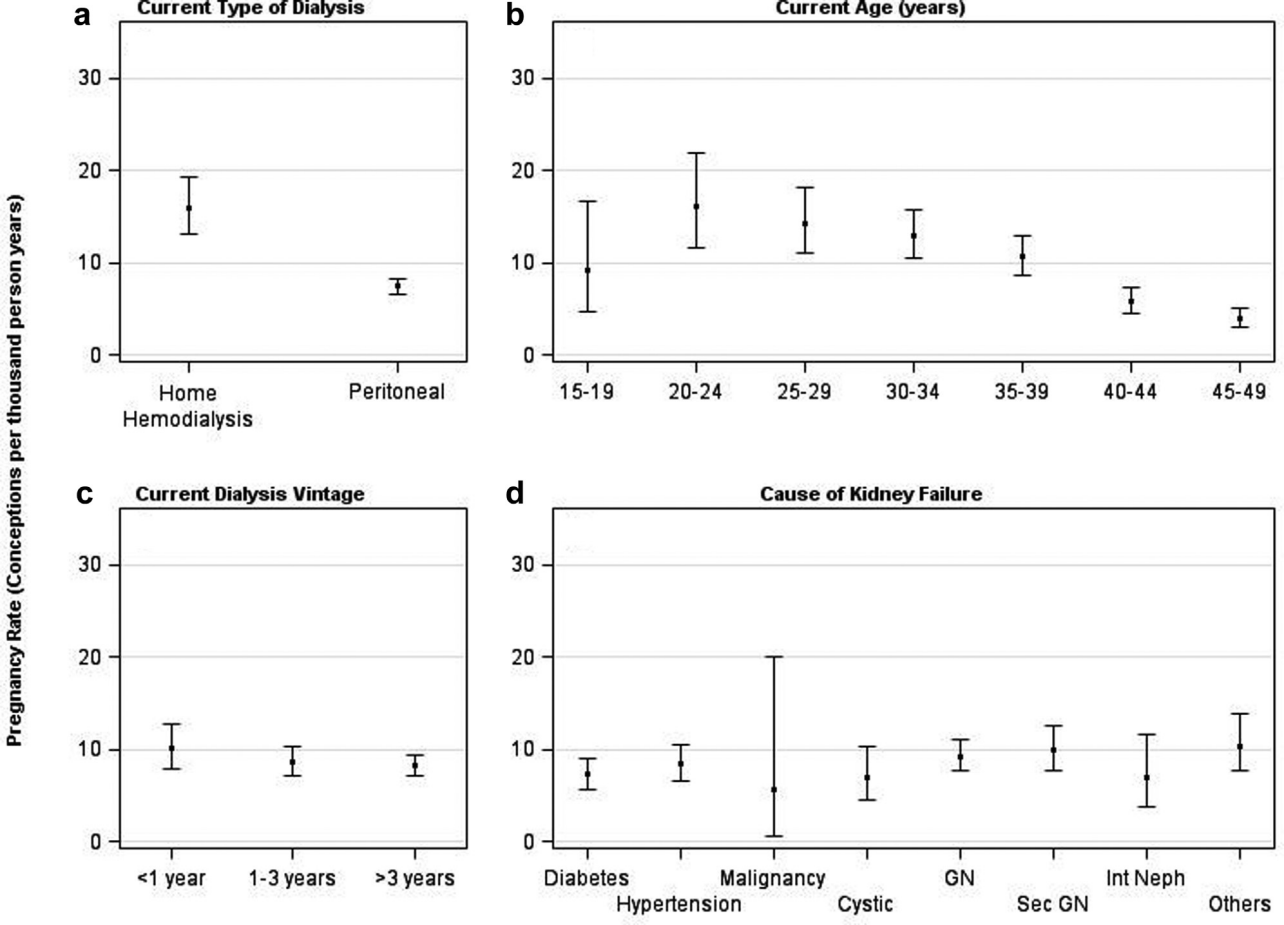
Pregnancy rates in women receiving home dialysis by (a) type of dialysis modality, (b) current age, (c) current dialysis vintage, (d) cause of kidney failure. GN, glomerulonephritis; Int Neph, interstitial nephritis; sec GN, secondary glomerulonephritis.

**Figure 3:**
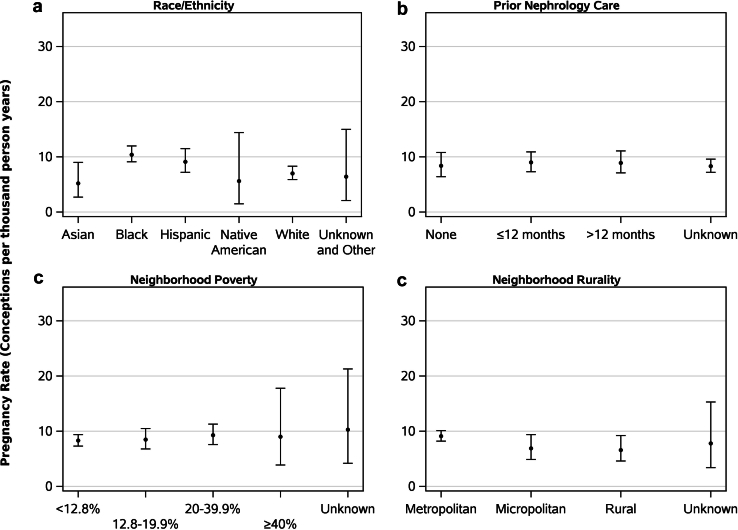
Pregnancy rates in women receiving home dialysis by (a) race/ethnicity, (b) previous nephrology care, (c) neighborhood poverty, (d) neighborhood rurality.

In Supplementary Table S1, we show the unadjusted rates of pregnancies by patient characteristics. Overall, the unadjusted rate of pregnancy was 8.6 PTPY (95% CI, 7.8–9.4). The rate of pregnancy was higher among women receiving home hemodialysis (16.0 PTPY; 95% CI, 13.1–19.2) than those receiving peritoneal dialysis (7.5 PTPY; 95% CI, 6.7–8.3) (Figure 2a). Regarding age, the pregnancy rate was highest in women aged 20 to 24 years (16.2 PTPY; 95% CI, 11.6–22.0), and lowest in women aged 45 to 49 years (3.9 PTPY; 95% CI, 3.0–5.1) (Figure 2b). Pregnancy rates were higher among women with dialysis vintage <1 year (10.1 PTPY; 95% CI, 7.9–12.8) than for those with dialysis vintage of 1 to 3 years (8.7 PTPY; 95% CI, 7.2–10.3) or >3 years (8.2 PTPY; 95% CI, 7.2–9.3) (Figure 2c). Regarding cause of kidney failure, pregnancy rates were higher among women with kidney failure due to secondary glomerulonephritis/vasculitis (9.9 PTPY; 95% CI, 7.7–12.6) and glomerulonephritis (9.2 PTPY; 95% CI, 7.7–11.0), and lower among women with kidney failure due to malignancy (5.6 PTPY; 95% CI, 0.7–20.1) (Figure 2d). With regard to race or ethnicity, the pregnancy rate was highest in Black women (10.4 PTPY; 95% CI, 9.1–12.0) followed by Hispanic (9.1 PTPY; 95% CI, 7.2–11.5), White (7.0 PTPY; 95% CI, 5.9–8.3), Native American (5.6 PTPY; 95% CI, 1.5–14.4), and Asian women (5.2 PTPY; 95% CI, 2.7–9.0) (Figure 3a). Regarding previous nephrology care, pregnancy rates were higher for women who received 0 to 12 months and >12 months of previous nephrology care than women who did not receive any previous nephrology care (Figure 3b). Pregnancy rates were higher in neighborhoods with 20.0% to 39.9% poverty (9.3 PTPY; 95% CI, 7.6–11.3) and ≥40% poverty (9.0 PTPY; 95% CI, 3.9–17.8) (Figure 3c). With regard to rurality, pregnancy rates were higher for women from metropolitan areas (9.1 PTPY; 95% CI, 8.2–10.1) than women from micropolitan (6.9 PTPY; 95% CI, 4.9–9.4) or rural (6.6 PTPY; 95% CI, 4.6–9.2) areas (Figure 3d).

### Factors Associated with Pregnancies on Home Dialysis

**Figure 4:**
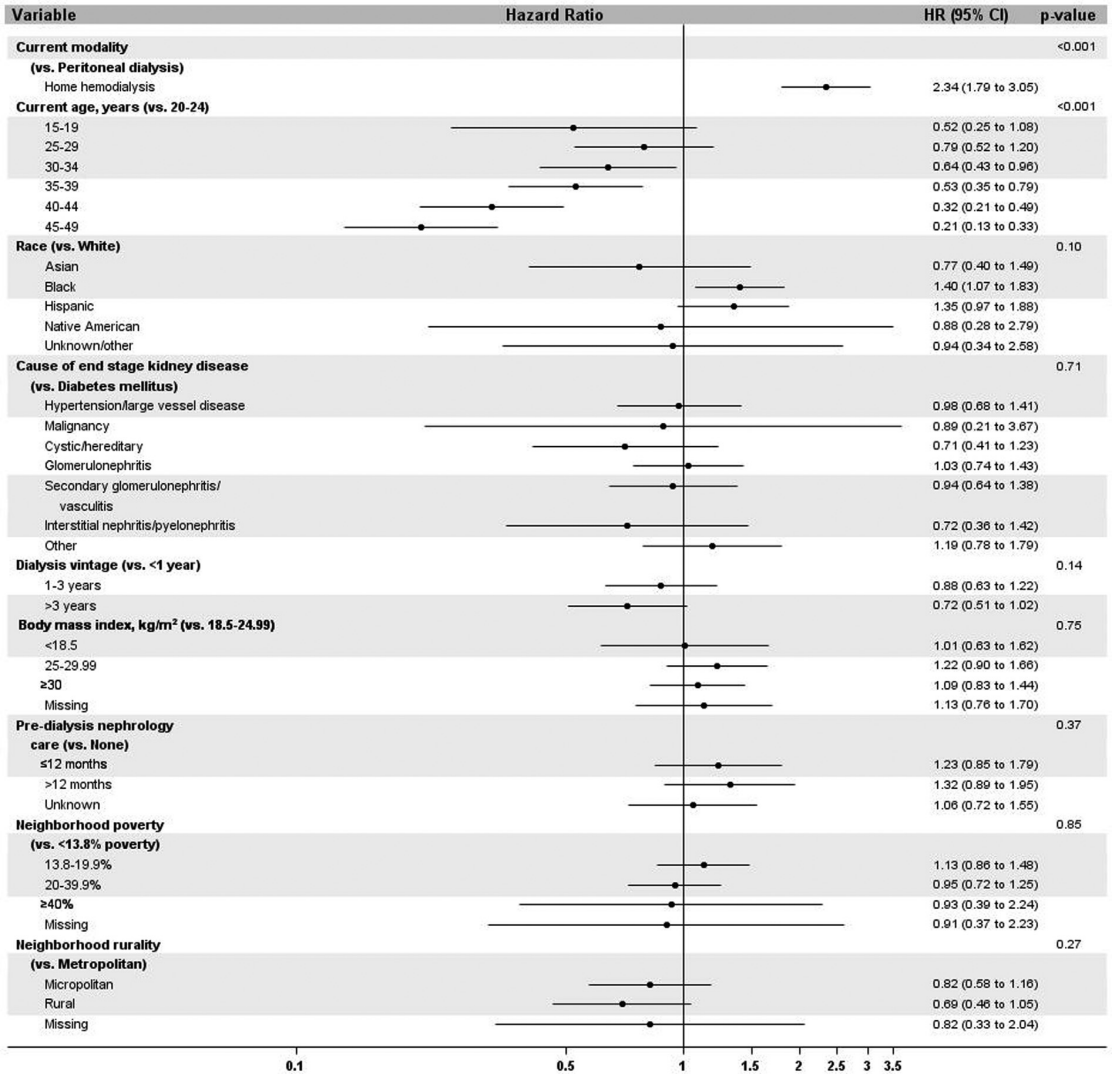
Main effects model showing factors associated with pregnancy in patients receiving home dialysis. CI, confidence interval; HR, hazard ratio.

In Figure 4, we show factors associated with the likelihood of pregnancy among patients receiving home dialysis from the adjusted time-to-event model. Women receiving home hemodialysis had a 2.3-fold higher likelihood of pregnancy than women receiving peritoneal dialysis (HR, 2.34; 95% CI, 1.79–3.05). Compared with women aged 20 to 24 years, the likelihood of pregnancy was lower in women aged 30 to 34 years (HR, 0.64; 95% CI, 0.43–0.96), those aged 35 to 39 years (HR, 0.53; 95% CI, 0.35–0.79), those aged 40 to 44 years (HR, 0.32; 95% CI, 0.21–0.49), and those aged 45 to 49 years (HR, 0.21; 95% CI, 0.13–0.33). Compared with White women, Black women had a higher likelihood of pregnancy (HR, 1.40; 95% CI, 1.07–1.83). There was no difference in the likelihood of pregnancy with regard to body mass index, cause of kidney failure, socioeconomic status, rurality, predialysis nephrology care, or time on dialysis.

### Fetal Outcomes

In Table 3, we show fetal outcomes by home dialysis modality. Overall, the following were the rates of fetal outcomes: live birth (32%, 2.8 PTPY), stillbirth (0.7%, 0.1 PTPY), spontaneous abortion (21.7%, 1.9 PTPY), therapeutic abortion (6.4%, 0.6 PTPY), ectopic or trophoblastic pregnancies (0.9%, 0.1 PTPY), and unknown outcome (38.2%, 3.3 PTPY). Live birth rates were higher in women receiving home hemodialysis than in those receiving peritoneal dialysis (36.7% vs. 30.5%, 5.9 PTPY vs. 2.3 PTPY). There were higher rates of unknown fetal outcomes in both women on home hemodialysis and those on peritoneal dialysis (26.6% vs. 42.1%, 4.2 PTPY vs. 3.1 PTPY)

**Table 3:** Rate of fetal outcomes overall and by dialysis modality

Fetal outcome		Overall	Home dialysis modality	
			Home hemodialysis	Peritoneal dialysis
Live births^a^	*n* (%)	140 (32.0)	40 (36.7)	100 (30.5)
	Rate (95% CI)	2.8 (2.3–3.3)	5.9 (4.2–8.0)	2.3 (1.9–2.8)
Stillbirths^a^	*n* (%)	∗	∗	∗
	Rate (95% CI)	0.1 (0.0–0.2)	0.3 (0–1.1)	0.0 (0.0–0.1)
Spontaneous abortions	*n* (%)	95 (21.7)	32 (29.4)	63 (19.2)
	Rate (95% CI)	1.9 (1.5–2.3)	4.7 (3.2–6.6)	1.4 (1.1–1.8)
Therapeutic abortions	*n* (%)	28 (6.4)	∗	∗
	Rate (95% CI)	0.6 (0.4–0.8)	0.9 (0.3–1.9)	0.5 (0.3–0.8)
Ectopic/trophoblastic pregnancies	*N* (%)	∗	∗	∗
	Rate (95% CI)	0.1 (0.0–0.2)	0.0 (0.0–0.5)	0.1 (0.0–0.2)
Unknown outcomes	*n* (%)	167 (38.2)	29 (26.6)	138 (42.1)
	Rate (95% CI)	3.3 (2.8–3.8)	4.2 (2.8–6.1)	3.1 (2.6–3.7)

CI, confidence interval.

Rate (95% CI) reported in per 1000 person-years.

a A pregnancy that results in both a live and a stillbirth would contribute to both categories

∗ Per Centers for Medicare and Medicaid cell suppression policy, cells that display individuals (n) from 1 to 10 or cells that allow a value of 1 to 10 derived from other reported cells are suppressed.

## Discussion

With 328 pregnancies on peritoneal dialysis and 109 pregnancies on home hemodialysis, our study is the first to report accurate pregnancy rates in the home dialysis population in the United States. To our knowledge, this is one of the first reports that examined the association of factors, including type of home dialysis modality, race or ethnicity, socioeconomic status, and rurality, with the likelihood of pregnancy in women on home dialysis.

Our study shows pregnancy rates of 8.6 PTPY in the prevalent home dialysis population. Although there are case reports and case series reporting pregnancies on home dialysis, little is known about pregnancy rates.^11^^,^^12^^,^^14^ A recent study showed a higher pregnancy rate of 17 PTPY in women with end-stage kidney disease undergoing dialysis; however, this included women on any dialysis modality, with the majority of them getting in-center hemodialysis.^23^ Oliverio *et al.*^2^ reported the unadjusted delivery rate of 3.8 PTPY in women with kidney failure undergoing in-center hemodialysis in the United States.^2^ Most earlier studies have reported much lower pregnancy rates in the dialysis population. For example, the Australian and New Zealand Dialysis and Transplant Registry reported a pregnancy rate of 2.1 PTPY in women undergoing dialysis from 1966 to 2008.^24^ The lower pregnancy rates were attributed to the use of voluntary registries, typical biases of surveys, and differences in the study methodology. Importantly, none of these studies looked at pregnancy rates, specifically in the home dialysis population.

Our study showed lower pregnancy rates in the home dialysis population than in the transplant population. Studies using the USRDS have reported a pregnancy rate of 13.8 PTPY (95% CI, 12.3–15.5) and a delivery rate of 4.6 PTPY among Medicare-insured kidney transplant recipients of childbearing age in the United States.^2^^,^^3^

Women receiving home hemodialysis, as compared to peritoneal dialysis, had a higher pregnancy rate and about a 2-fold higher probability of conceiving. Although no studies have compared the pregnancy rates by type of home dialysis modality, our results are consistent with the previous literature showing lower conception rates for women undergoing peritoneal dialysis. The ANZDATA Registry from Australia and New Zealand reported pregnancy rate of 1.1 PTPY on peritoneal dialysis, and another study from the United States reported a delivery rate between 0.5 to 1.4 PTPY (2002–2015) on peritoneal dialysis.^2^^,^^24^ Women on peritoneal dialysis have about 50% lower likelihood of pregnancy than on hemodialysis.^3^ The reasons for the lower conception rates in women on peritoneal dialysis versus home hemodialysis remain unclear, especially because patients on peritoneal dialysis have a higher residual renal function.^25^ It is postulated that indwelling intraperitoneal solutions can interfere with the transit and implantation of the ovum to the uterus.^26^ Peritonitis during pregnancy with peritoneal dialysis is associated with premature rupture of membranes, chorioamnionitis, and postpartum hemorrhage.^27^ Intensification of dialysis to maintain lower blood urea nitrogen <40 mg/dl to improve fetal outcomes is usually not achievable with peritoneal dialysis, especially with uterine distention after the first trimester. Therefore, it is plausible that home hemodialysis is the preferred mode of home dialysis therapy over peritoneal dialysis in women contemplating pregnancy.

The current study shows racial or ethnic disparities in the occurrence of pregnancy on home dialysis, with Black women having a 40% higher likelihood of pregnancy than White women. Although there is no literature, to the best of our knowledge, examining racial or ethnic disparities in pregnancy specifically on home dialysis, higher pregnancy rates have been reported in Black women on dialysis, and the present study may be mirroring the trend.^3^ More research is needed to understand the racial or ethnic differences in pregnancy on home dialysis.

A significant strength of our study is that it studied pregnant women, specifically those receiving home dialysis in the United States, thus providing us with information about pregnancy rates for a heterogeneous population. In addition, we identified specific factors that increase the likelihood of pregnancy that may require consideration while counseling and managing pregnancy in women on home dialysis. This may further help in making guidelines for the management of pregnancy among women undergoing home dialysis. The limitations of our study include the observational design, which precludes the determination of causality. In addition, variability in the quality and completeness of the data recorded on form 2728 is associated with its inherent limitations for utilizing USRDS data. Patient-level variables of health literacy, contraceptive use, and use of assisted reproductive techniques, which may impact pregnancy, are not captured and were not available for our analysis. Although the rate of live births was higher in the home hemodialysis pregnancies than the peritoneal dialysis pregnancies, an unknown pregnancy outcome in approximately 40% of the cohort remains another limitation. However, our study accounts for all the pregnancies, and not just those reported in the voluntary registries or single centers, thereby predicting accurate pregnancy rates among women on home dialysis.

In conclusion, our study demonstrates higher pregnancy rates on home dialysis and significant differences in rates by type of home dialysis modality and race or ethnicity. Currently, there are no concerted efforts that guide us in management and counseling of pregnancy in women on home dialysis. Crafting successful solutions requires strategies to improve access to care for pregnant women on home dialysis, and physician and patient education about their practice patterns and possible biases. Information from the present study will guide health care providers in counseling and shared decision-making regarding reproductive health. This may further lead to policy changes and potentially deliver customized care based on the probability of pregnancy in the home dialysis population.

## Disclosure

SS is the associate editor of Advances in Kidney Disease and Health journal and has received an honorarium from the Bayer pharma and the Vifor pharma outside the submitted work. JP reports personal fees from Baxter; personal fees from FMC; grants from AHRQ; personal fees from Arbor Research; personal fees from Bayer / GSK / Otsuka / AZ/ US Renal Care, from ARA; personal fees from Outset Medical; and personal fees from iRen Medical, outside the submitted work. EW reports current employment at DaVita Clinical Research and past employment at Satellite Healthcare, outside the submitted work; and Volunteer Director of Medical Education Institute (MEI). All the other authors declared no competing interests.

## Supplementary Material

Supplementary File (PDF)

Discharge diagnoses and medical procedures indicative of pregnancy.

**Figure S1.** The stepwise approach of the methodology to identify pregnancies.

**Table S1.** Unadjusted pregnancies rates per 1000 person-years in women with kidney failure on home dialysis.
