# Supplementary material for: Pregnancies in Women With Kidney Failure on Home Dialysis in the United States
Source: Kidney Int Rep. 2024 Feb 1;9(4):907–18. doi: 10.1016/j.ekir.2024.01.045 (PMC11101807; doi:10.1016/j.ekir.2024.01.045)
Supplement: Supplementary File (PDF) [file mmc1.pdf]

Discharge diagnoses and medical procedures indicative of pregnancy

Live Birth

ICD-9 diagnoses: 641.01, 641.11, 641.21, 641.31, 641.81, 641.91, 642.01, 642.02, 642.11, 642.12, 642.21, 642.22, 642.31, 642.32, 642.41, 642.42, 642.51, 642.52, 642.61, 642.62, 642.71, 642.72, 642.91, 642.92, 643.01, 643.11, 643.21, 643.81, 643.91, 644.2, 645.01, 645.11, 645.21, 646.11, 646.12, 646.21, 646.22, 646.31, 646.41, 646.42, 646.51, 646.52, 646.61, 646.62, 646.71, 646.81, 646.82, 646.91, 647.01, 647.02, 647.11, 647.12, 647.21, 647.22, 647.31, 647.32, 647.41, 647.42, 647.51, 647.52, 647.61, 647.62, 647.81, 647.82, 647.91, 647.92, 648.01, 648.02, 648.11, 648.12, 648.21, 648.22, 648.31, 648.32, 648.41, 648.42, 648.51, 648.52, 648.61, 648.62, 648.71, 648.72, 648.82, 648.91, 648.92, 650, 651.01, 651.11, 651.21, 651.31, 651.41, 651.51, 651.61, 651.81, 651.91, 652.01, 652.11, 652.21, 652.31, 652.41, 652.51, 652.61, 652.71, 652.81, 652.91, 653.01, 653.11, 653.21, 653.31, 653.41, 653.51, 653.61, 653.71, 653.81, 653.91, 654.01, 654.02, 654.11, 654.12, 654.21, 654.31, 654.32, 654.41, 654.42, 654.51, 654.52, 654.61, 654.62, 654.71, 654.72, 654.81, 654.82, 654.91, 654.92, 655.01, 655.11, 655.21, 655.31, 655.41, 655.51, 655.61, 655.71, 655.81, 655.91, 656.01, 656.11, 656.21, 656.31, 656.51, 656.61, 656.71, 656.81, 656.91, 657.01, 658.01, 658.11, 658.21, 658.3, 658.30, 658.31, 658.33, 658.41, 658.81, 658.91, 659.0, 659.00, 659.01, 659.03, 659.1, 659.10, 659.11, 659.13, 659.2, 659.20, 659.21, 659.23, 659.3, 659.30, 659.31, 659.33, 659.41, 659.51, 659.61, 659.71, 659.8, 659.80, 659.81, 659.83, 659.9, 659.90, 659.91, 659.93, 660, 660.0, 660.00, 660.01, 660.03, 660.1, 660.10, 660.11, 660.13, 660.2, 660.20, 660.21, 660.23, 660.3, 660.30, 660.31, 660.33, 660.4, 660.40, 660.41, 660.43, 660.5, 660.50, 660.51, 660.53, 660.6, 660.60, 660.61, 660.63, 660.7, 660.70, 660.71, 660.73, 660.8, 660.80, 660.81, 660.83, 660.9, 660.90, 660.91, 660.93, 661, 661.0, 661.00, 661.01, 661.03, 661.1, 661.10, 661.11, 661.13, 661.2, 661.20, 661.21, 661.23, 661.3, 661.30, 661.31, 661.33, 661.4, 661.40, 661.41, 661.43, 661.9, 661.90, 661.91, 661.93, 662, 662.0, 662.00, 662.01, 662.03, 662.1, 662.10, 662.11, 662.13, 662.2, 662.20, 662.21, 662.23, 662.3, 662.30, 662.31, 662.33, 663, 663.0, 663.00, 663.01, 663.03, 663.1, 663.10, 663.11, 663.13, 663.2, 663.20, 663.21, 663.23, 663.3, 663.30, 663.31, 663.33, 663.4, 663.40, 663.41, 663.43, 663.5, 663.50, 663.51, 663.53, 663.6, 663.60, 663.61, 663.63, 663.8, 663.80, 663.81, 663.83, 663.9, 663.90, 663.91, 663.93, 664, 664.0, 664.00, 664.01, 664.1, 664.10, 664.11, 664.2, 664.20, 664.21, 664.3, 664.30, 664.31, 664.4, 664.40, 664.41, 664.5, 664.50, 664.51, 664.8, 664.80, 664.81, 664.9, 664.90, 664.91, 665, 665.0, 665.00, 665.01, 665.03, 665.1, 665.10, 665.11, 665.2, 665.20, 665.22, 665.3, 665.30, 665.31, 665.4, 665.40, 665.41, 665.5, 665.50, 665.51, 665.6, 665.60, 665.61, 665.7, 665.70, 665.71, 665.72, 665.8, 665.80, 665.81, 665.82, 665.83, 665.9, 665.90, 665.91, 665.92, 665.93, 666, 666.0, 666.00, 666.02, 666.1, 666.10, 666.12, 667, 667.0, 667.00, 667.02, 667.1, 667.10, 667.12, 668, 668.0, 668.00, 668.01, 668.02, 668.03, 668.1, 668.10, 668.11, 668.12, 668.13, 668.2, 668.20, 668.21, 668.22, 668.23, 668.8, 668.80, 668.81, 668.82, 668.83, 668.9, 668.90, 668.91, 668.92, 668.93, 669, 669.0, 669.00, 669.01, 669.02, 669.03, 669.1, 669.10, 669.11, 669.12, 669.13, 669.2, 669.20, 669.21, 669.22, 669.23, 669.3, 669.30, 669.32, 669.4, 669.40, 669.41, 669.42, 669.43, 669.5, 669.50, 669.51, 669.6, 669.60, 669.61, 669.7, 669.70, 669.71, 669.8, 669.80, 669.81, 669.82, 669.83, 669.9, 669.90, 669.91,

Supplementary Figure S1. The stepwise approach of the methodology to identify pregnancies

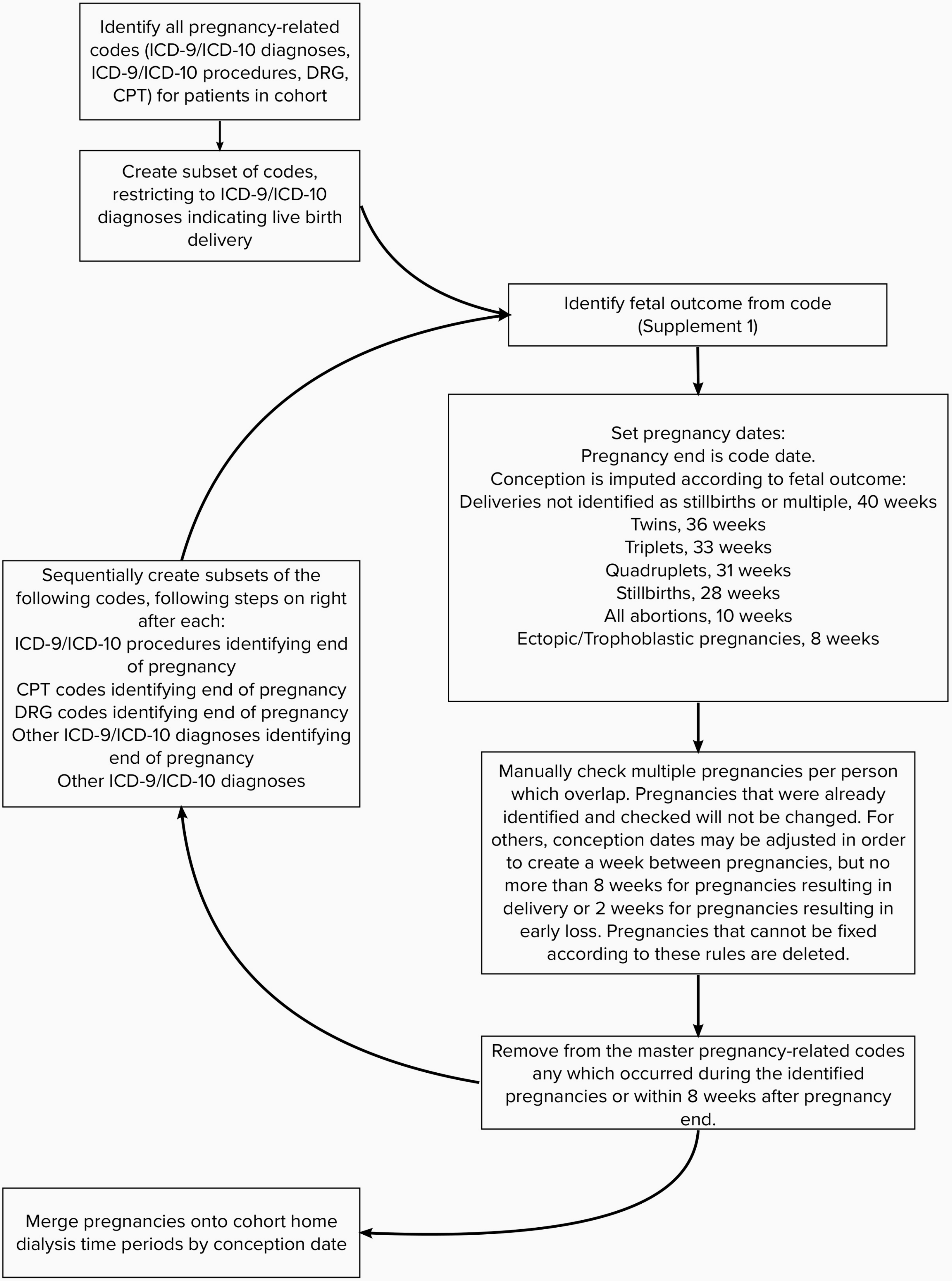

Supplementary Table S1. Unadjusted pregnancy rates per 1000 person-years in women with kidney failure on home dialysis

	Rate[a] (95% CI)
Overall	8.6 (7.8-9.4)
Modality	
Home hemodialysis	16.0 (13.1-19.2)
Peritoneal dialysis	7.5 (6.7-8.3)
Age (years)	
15-19	9.3 (4.6-16.6)
20-24	16.2 (11.6-22)
25-29	14.3 (11.2-18.1)
30-34	12.9 (10.4-15.8)
35-39	10.7 (8.7-13)
40-44	5.8 (4.5-7.3)
45-49	3.9 (3.0-5.1)
Dialysis vintage	
<1 year	10.1 (7.9-12.8)
1-3 years	8.7 (7.2-10.3)
>3 years	8.2 (7.2-9.3)
Cause of kidney failure	
Diabetes mellitus	7.3 (5.7-9.1)
Glomerulonephritis	9.2 (7.7-11)
Secondary glomerulonephritis/vasculitis	9.9 (7.7-12.6)
Interstitial nephritis/pyelonephritis	6.9 (3.8-11.7)
Hypertension/large vessel disease	8.4 (6.7-10.4)
Cystic/hereditary/congenital	7.0 (4.5-10.4)
Malignancy	5.6 (0.7-20.1)
Others	10.4 (7.6-13.8)
Race/ethnicity	
Asian	5.2 (2.7-9)
Black	10.4 (9.1-12)
Hispanic	9.1 (7.2-11.5)
Native American	5.6 (1.5-14.4)
White	7.0 (5.9-8.3)
Unknown/Others	6.4 (2.1-15)
Prior nephrology care	
None	8.4 (6.4-10.8)
≤ 12 months	9.0 (7.3-10.9)
>12 months	8.9 (7.1-11.1)
Unknown	8.3 (7.2-9.6)
Neighborhood poverty	
< 12.8%	8.3 (7.3-9.4)
12.8-20%	8.5 (6.8-10.5)
20-39.9%	9.3 (7.6-11.3)
≥40%	9.0 (3.9-17.8)
Unknown	10.3 (4.2-21.3)
Neighborhood rurality	
Metropolitan	9.1 (8.2-10.1)

Micropolitan	6.9 (4.9-9.4)
Rural	6.6 (4.6-9.2)
Unknown	7.8 (3.4-15.3)

[a]Rate reported in per 1000 person-years.
